# Extra Supplement—The Nursing Mirror

**Published:** 1889-06-08

**Authors:** 


### The Nursing Mirror.

All Contributions for this Supplement should be addressed "The Nursing Mirror," care of the Editor, 139-140, Salisbury Court, Fleet Street, London, E.C.

### En Passant.

PRACTICAL APPRECIATION.—The doctors of Queenstown General Hospital having signified to the committee their entire satisfaction with the very efficient manner in which the head and assistant nurses discharged their duties, the House Committee recommend that they receive an annual increase to their salaries of £10 and £2 respectively.

BREAKFAST BASKETS.—In the current number of *Work and Leisure* there is a short paper on "Invalid Breakfast Baskets." It seems that these baskets can be ordered at varying prices, and contain different dainties, according to the season. One of the most trying parts of nursing in a far country village is the difficulty in procuring variety in the food. Many a nurse, after her three years in hospital, goes forth to private nursing little knowing the difficulties before her. Unless she has a true feminine knowledge of cookery and dishing-up, and is familiar with old-fashioned recipes for cooling drinks and herb teas, she will find herself at a great loss. Even dainties sent by post are not much use unless properly cooked and prettily served, and seasoned with bright conversation. Still, it is well for nurses to remember the conveniences of the parcel post; then, if they find themselves in an out-of-the-way spot, they can order an invalid breakfast basket to be sent them three times a week.

EXAMINATION QUESTIONS.—The matron of a Scotch hospital writes to know if we could not give surgical and medical questions for prize competitions instead of giving puzzles. The idea is a good one, and could doubtless be carried out with the help of some of our correspondents if they would interest themselves in the matter. We have questions sent us now, but no one has volunteered to send us answers. Most nurses are too much engaged with their own annual examinations and weekly classes to try for external competitions; but still in those country and cottage hospitals where examinations are unknown and work often slack, candidates might be found anxious to improve their minds by answering prize queries. We fancy it must be, indeed, a lonely spot where the matron who makes the suggestion resides, for she also sends us the following story: "A boy was brought to our hospital with his leg completely jagged off. A gentleman who had seen the accident came to enquire for him, and asked if the doctors had put the leg on again!"

NURSING LECTURES.—The woman who is ignorant of the first principles of nursing, such as how to change sheets or chart a temperature, will soon be a creature of the past. It seems almost impossible to take up a provincial paper without finding some record of lectures on nursing, given either by a trained nurse or some general practitioner. At the termination of Dr. Drury's course of lectures on "Nursing," lately given at Halifax, under the auspices of the St. John Ambulance Association, an examination was conducted by Dr. A. G. Barrs, physician to the Leeds Infirmary. All the candidates who entered were successful. At the last lecture of the course, the secretary (Miss Ellie Illingworth), on behalf of members of both the "First Aid" and "Nursing" classes, presented Dr. Drury with a choice selection of books, viz.: George Eliot's works, in eight handsomely bound volumes, including the life of George Eliot, also a case containing Shakespeare's works, in twelve volumes, as some acknowledgment of the time and trouble he had expended in giving the lectures.

WHERE GIFTS SHOULD GO.—A lady at Southport, whose servant had been kindly treated at Manchester Royal Infirmary, has sent a present of twenty-five guineas to the infirmary. Mr. Whitehead, hon. surgeon, writes to suggest that the money be allowed to form the nucleus of a superannuation fund for the nurses, and offers to add another twenty-five guineas himself if this is done. May we suggest to Manchester Royal Infirmary authorities that if they would affiliate themselves to the Pension Fund, they would do the best thing possible for all their nurses? Half measures are a mistake in money matters; and fifty guineas won't go far unless joined to some central scheme.

THE CARE OF THE INSANE.—We do not wish our friends in asylums to think we are forgetting them, therefore we gather a few notes from a very interesting book by Dr. Charles Mercier on "The Nervous System and the Mind." A lunatic is not the less a lunatic because he is able to work out the binomial theorem, or to draw up the plans and specifications and oversee the work of building a church. Many idiots have considerable skill in music, and some have exceptional powers of calculation. Every asylum contains lunatics who are skilled workmen at some handicraft or other, and may contain, as patients, artists of no mean capacity. The possession of any or all of the three faculties of originality, cleverness, and skill is quite consistent with insanity, for they may yet be unable to "manage their affairs." So long as a man can gain his own living, however stupid he may be—he is not imbecile. Insanity consists in disorder of the process of adjustment of the organism to its environment. In Jacksonian epilepsy a fit begins in the thumb and index finger; the spasm there becomes stronger, the other fingers become convulsed, movement at the wrist is added, then at the elbow, and then at the shoulder.

A WARNING FOR INSTITUTIONS.—Dr. Lake, of Southampton, attended a case nursed by a nurse from the Hampshire Nurses' Institute. Dr. Lake alleges that this nurse slandered him to his patient, and that when he complained to the committee of the institute they permitted the nurse to resign, instead of making full enquiries. Dr. Lake has put the whole affair before the public in the local press, and gives the following hints which may be useful to others: "I claim that when any charge of a serious nature is made by a responsible person against an institution nurse, it is the duty of the committee themselves to investigate the matter and take every pains to prove its truth, and not the position taken up by that committee, that they sit as judges and are called on to ignore anything not proved by strict legal evidence. The throwing the whole duty of investigation on the informant, and holding *in terrorem* the intensely disagreeable task to any lady of appearing and facing the nurse, probably supported by a trained lawyer, is, to my mind, monstrous. The nurse is their servant, they have a duty to the patient and to the doctor, as well as to their servant, the nurse. It never entered into my head to think that the committee would discharge a nurse on the unproved dictum of a medical man; but I do say that the charge by a doctor of serious fault on the part of a nurse demands an immediate and exhaustive investigation by the committee. Secondly, I consider that when a serious complaint is made by a responsible person, and especially by a medical man—who owes a serious duty towards his patient—against a nurse, she should be suspended from work till her character is cleared, and not sent out to case after case as though she were a perfect nurse."

### A First Experience.

IN TWO PARTS.—PART I.

ALL my life I had had a very great wish to learn nursing in a hospital, and some years ago my wish was gratified. I thought in London I should probably get better training than in the provinces, so after obtaining particulars I finally chose a hospital where I could be taken at once as a paying probationer. My credentials being approved of by the matron, materials were sent for my uniform, and I was told to come to the hospital between five and six on a certain day. My home being in the country, I spent the night before at the house of an old friend living in Bayswater, and at twenty-five minutes to six on the evening agreed upon I arrived at the gates of the hospital.

As it was late in the autumn it was quite dark, and pouring with rain. I dismissed my cab and walked through the gate, but was immediately stopped by the porter, who called out, "Hi! what do you want?" I answered in rather a nervous voice, "I am a new probationer." He said, "All right," and I passed on across an open space and up some wide stone steps into an entrance-hall. I looked about for a minute, not knowing which way to turn. Presently a porter crossed the hall. I went up to him, told him who I was, and asked where I had better go. He answered, "Go to the matron's office." I said, "Where is it?" "Down that passage to the left." Accordingly, I went down the passage, and knocked at a door with "Matron's Office" painted on it; but knocking had no effect, and I was wondering what I should do next, when a nurse came by, and said, "What are you doing there?" I told her I wanted to see the matron. "Matron's gone home hours ago," was her answer. "Then, will you kindly tell me where I am to go?" I said. "Yes, go to the Nurses' Home," and she pointed to a door opposite to the one I had entered the hospital by, leading into what looked like a London square. It was pitch dark, and I had no idea in which direction the home was. At that moment another nurse came running in out of the rain, and I said, "Would you mind telling me where the Nurses' Home is?" She said, "Do you see some white steps there? That is it." After looking steadily in the direction she pointed for a minute, I could just make out some steps in the distance, and I stepped out into the wet and dark once more, walked across the square and up the steps, and rang the door-bell. But ringing the bell seemed no more effectual than knocking at the matron's door. I must have stood there in the rain for quite ten minutes when a nurse came out, and once more I asked where I was to go. She told me to go down a passage and I should see a door with "Home Sister" on it. I knocked at this door, and was told to come in, I went in, and explained to the sister, whom I will call Miss Green, who I was. She took me at once through many passages and up many stairs to my bedroom.

It was now about six o'clock. Miss Green said: "Supper is at half-past nine; you must come in uniform." I told her my luggage had not come. She said, "Well, you must not come out of uniform." She then left me. Fortunately, I had some knitting and a book, so I managed to occupy myself till nine o'clock, when, to my great relief, a porter came to my room, bringing my box, so I quickly unpacked and arrayed myself in my uniform. Presently, there was a knock at my door, and someone said, "Miss Green says you are to come to supper." I went down a long passage till I came to the dining-hall. I opened the door and went in, but not one vacant seat could I see, and I think everyone of those 100 nurses stopped eating and looked at me as I stood just inside the door, till at last one of them said sharply, "Come, nurse, don't you want any supper?" "Yes," I said, "if I could only find a seat." "There's one over there," she said. So I made my way to this seat and sat down, feeling very shy and uncomfortable, and a little inclined to cry. A nurse opposite me said, "Is this your first night here?" I told her it was. She said, "Ah, then, I pity you; you have got something to go through!" This did not make me feel much more comfortable.

At six o'clock the next morning, I was awoke by a tremendous banging at my door, and in great haste I got up and dressed, and made my way to the dining-hall, where we had breakfast, which that morning consisted of two sardines each, as much bread as we liked, and tea, and coffee and milk. After breakfast, the sister who was present asked me what my name was, and why I had not given her my number as I came in. I told her that I did not know I had a number. She then told me which ward I was to go to, and sent a nurse to show me the way. When we got there, the nurse in charge said, "Is that new probationer for me?" The other nurse told her, "Yes"; she began to grumble, and said, "That's just my luck. I am always having the new probationers shoved off on me. It's too bad," etc. I thought to myself, it is evident I am not wanted here. I had better have stayed at home.

It was a female medical ward, with twenty beds in it, all full but three. The nurse told me to help her make the beds, which I did, then we swept the floor, afterwards I polished some tins, dusted the ward, etc. I found my nurse was not half so disagreeable as she appeared at first. I soon got to like her very much.

About nine o'clock the sister who superintended the ward came and spoke to me, and told me to go off duty from ten till twelve, and to report myself to the matron in that time, which I did; but she kept me waiting three-quarters of an hour before she could see me, which diminished my time off duty. She shook hands with me, and said she hoped I should be comfortable. Then I went to my room, finished my unpacking, tidied my room, and wrote a letter. Then it was time to go on duty again. Directly after I got into the ward the dinners were brought up. The sister carved the meat; the probationers carried the plates to the different patients. After the dinners were over I washed the knives and forks, swept up the crumbs, and put the beds straight, whilst the nurse went to her dinner; when she came back I went to mine. By this time I had been given a number, which I did not fail to tell the sister as I came into the dining-hall. After dinner, when I went back to my ward, the charge nurse went off duty from two o'clock till four, and I was left in charge. There was nothing much to do, as most of the patients were sleeping, so I got my knitting and sat down at the nurses' table, very glad to rest for a little while. When I had been sitting there about half an hour, the sister came in with one of the visiting physicians, the house physician, and about a dozen students. I did not see that there was anything for me to do, so I went calmly on with my knitting. Presently sister beckoned me to her, and said very gravely, "Nurse, you must never remain seated when the doctors are in the ward, but must go round with them." They stayed a long time, the visiting physician giving a little lecture on each patient to the students. I got very tired with standing, and presently began to feel dreadfully faint. The sister was very kind, and let me go away and sit down by an open window till I felt better. When the doctors had gone it was time to get the teas, which I did with the help of a convalescent patient. When the teas were finished I washed up the tea-things, put them away, and swept up the crumbs. Then the charge nurse came back, and she and I had our own tea in the ward. After that the beds had to be made, the temperatures taken, and the ward made ready for the night, which took us till eight o'clock, when the gas was turned down to a spark, and the work was finished for that day, and nurse and I were only too glad to be able to sit quietly and rest till the night nurses came on duty at nine, and we went to supper, and then to bed.

*(To be continued.)*

### Nurses and Gossip.

"LET everything which you learn in the capacity of nurse in any household meet its tomb in your ear! Tell nothing good or bad concerning families in which you serve; for if you talk of the good, the mention of the bad will assuredly follow, even as 'the sparks fly upward.' In times of illness the secrets of a family cannot be hidden from your ken; the closet doors are open, and the eyes of the midnight-watching nurse cannot fail to see the skeletons which hide within. Can you suggest to me anything more contemptible, more deeply sunk beneath the dignity of womanhood, more repulsive to propriety, than the treacherous exposure of the poor bleeding wounds of sorrowing households to the public gaze and to the gossip's tongue?"—*Dr. Gaillard Thomas.*

### An Evening at the Grosvenor.

"April showers bring May flowers," runs the proverb, and we were reminded of it at the annual gathering of the Workhouse Infirmary Nursing Association in the Grosvenor Gallery, on the evening of the 20th of May.

The room and tea tables were literally covered with rhododendrons, lilacs, lilies, all the harbingers of summer mingled with hot-house flowers and fern, and later, when the bountiful tea, provided by the kindness of Lady Colebrooke, had been done full justice to, each of the nurses present received one, and in most cases two large bouquets to carry away with her.

Many members of the association were present, including Lady Brownlow, Lady Colebrooke, Lady Camilla Fortescue, Lady Knutsford, Lady Montagu, Hon. Mrs. Hardcastle, Hon. Mrs. Talbot, Mrs. Bonham Carter, Mrs. Lawrie, Mrs. Powell, Miss L. Twining, Miss Palmer, Miss Wood, and Miss Wilson (hon. sec.), Sir E. Sieveking, Dr. Symes Thompson, and Mr. Benton. Miss Sieveking, Miss J. Harrison, and many other ladies lent their aid; also Miss Hughes, Miss Moir, Mrs. Perry, and Miss Styring, all matrons of infirmaries.

Tea over, all adjourned to the large gallery, where Lady Colebrooke awarded medals and gratuities to twenty nurses, several of whom had remained for eight years at their several posts, and later in the evening the party again assembled in the same place to hear a few words from Lady Montagu on the subject of Indian hospitals. Lady Montagu has only just returned from the East, and her information was so easily given, so simply and graphically told, that the interested faces of her listeners showed they thought, as we did, that the address was but too quickly concluded.

From seven o'clock till past ten, the galleries presented an animated scene. The nurses, some seventy or eighty in number, were scattered about in twos and threes, many looking at the pictures, catalogue in hand, others chatting to sister nurses and friends not seen since the previous year. When the last one had left, the rooms looked and felt quite deserted without the neat figures, their white caps and aprons half hidden behind the masses of flowers they carried.

Nearly all present being either associates or friends, little reference was made to the association itself; but we cannot refrain from drawing attention to the admirable work it promotes—viz., the better nursing of the sick poor in our workhouse infirmaries. Quietly and steadily during the last ten years, the association has pushed its way, winning the approbation and respect of medical men and boards of guardians, and now so constant are the requests for its trained nurses, that the demand far exceeds the supply. The only limit to its further extension is the urgent want of funds for providing the training of a larger number of women.

### Recompense.

Oh deem not they are blest alone
  Whose lives a peaceful tenor keep;
The Power who pities man hath shown
  A blessing for the eyes that weep.

The light of smiles shall fill again
  The lids that overflow with tears;
The weary hours of woe and pain
  Are promises of happier years.

There is a day of sunny rest
  For every dark and troubled night;
And grief may hide an evening guest,
  But joy shall come with early light.

For God hath marked each sorrowing day,
  And numbered every secret tear;
And heaven's long age of bliss shall pay
  For all His children suffer here.

W. C. Bryant.

### The Nurse's Bookshelf.

### SPEAKING PLAINLY.*

There has been great constraint in nursing circles about the British Nurses' Association. Many women have felt deep disapproval of its schemes, but have preferred to keep silence as long as possible, lest outsiders should cry out once more at feminine bickerings and quarrels, and laugh at the lack of power of combination, which makes the weaker sex ridiculous. It needs great moral courage and firm faith, in truth, to criticise a movement started by our fellow-sisters, and all honour is due to Miss Lückes, matron of the London Hospital, for coming forward to represent the unspoken thoughts of the greater part of the nursing world. In a small pamphlet the reasons why nurses should not join this much-advertised association are plainly and simply put forth. There is no use of invective, no advancing of an opposition scheme; it is merely one woman begging others to look before they leap, and not to rashly take a step they will probably regret. The following are Miss Lückes' three objections to the British Nurses' Association: (1) The scheme of registration would hinder rather than advance a high standard of nursing, and would tend to prevent the progress and development of the work on its present lines. (2) It would inevitably injure the position which a well-trained nurse now holds in the estimation of the public, by rendering it increasingly difficult to distinguish between first and second-rate qualifications, and it is obvious that those who have most to lose will suffer most by an attempt to reduce all qualifications to a dead level of uniformity. (3) Of the minor objects of the British Nurses' Association, the establishment of convalescent homes for nurses, and of offices to facilitate the obtaining of engagements, are not needed, and if instituted would not be conducive to the welfare and advancement of the best nurses. These objections are enlarged on, and we are sure no-one can read the pamphlet dispassionately and not agree with the conclusions of the writer. The voice of authority has now pronounced the verdict on the British Nurses' Association.

### Everybody's Opinion.

[*Correspondence on all subjects is invited, but we cannot in any way be responsible for the opinions expressed by our correspondents. No communications can be entertained if the name and address of the correspondent is not given, or unless one side of the paper only be written on.*]

### ZENANA MEDICAL COLLEGE.

Miss Robinson writes: It will interest those friends who have so kindly and liberally responded to our appeal for funds to enable us to give free scholarships to two Syrians, pupils of a former student, to know that the following resolution was sent to Miss Proctor last month by the committee: "That one Syrian student, to be nominated by Miss Proctor, be given two years' free training, provided Miss Proctor undertakes the expenses of travelling, clothing, and all the usual extra or personal expenses; and that the committee will continue to make appeals for funds for the second scholarship." The following reply has just been received from Miss Proctor: "Mission School, Schwifat Beyrout, May 12th. Dear Dr. Griffith,—Your letter of April 25th, with the copy of the resolution passed at your committee meeting, has afforded me great pleasure, and I most heartily thank you for the efforts you have made on behalf of my pupils. They will be ready to come, I hope, next spring." The closing sentence in the letter makes us feel how important it is for us to

* What will trained nurses gain by joining the British Nurses' Association?" By Eva C. E. Luckes. J. and A. Churchill. Price 2d.

work while the door of opportunity is still open. "I am thankful to say we have not as yet been troubled by the Government, and I hope we may escape; but we rejoice with trembling as we know how our brethren around us are suffering." How soon the door may be closed we know not; shall we not do our utmost to enter it while we may? The committee desire to inform their kind friends and subscribers that the annual meeting will be held in the autumn (D.V.), and not as heretofore in June.

### A NURSES' CLUB.

Nurse Ellen Jay writes: Some time ago there was a proposal in The Hospital to establish a central room, where nurses could meet their friends, write letters, and borrow technical books. In fact, a room where the nucleus of a nurses' club could be formed. Now there is a nurses' club in Buckingham-street, but it has no room of its own to which members can retire at all times, but only borrows a room one evening a week to hold meetings and lectures. Could not the club in Buckingham-street affiliate itself to the club proposed by The Hospital, and thus secure a local habitation? The scheme is such a good one, and would be welcome to so many nurses, that it will be a pity if it is never carried out. Yet nurses must not be asked for a large subscription. About five shillings a year would be all they could afford, and I feel this would not pay for the hire of a room, unless all London nurses joined *en masse.* And nurses do not seem inclined to work together in large bodies; still if the room were used for two or three of the present small societies—such as the Guild of St. Barnabas, etc., it ought to secure prosperity. The Somerville Club for Women in Oxford-street is flourishing, and that has very wisely taken up its abode over an ærated bread shop, where cheap refreshment of a wholesome kind can be had. There is a speaking-tube from the clubroom down to the shop, and the girls bring up any food ordered. This is a notion worth considering when the choice of a clubroom for nurses has to be made, as I sincerely hope it will soon have to be.

### THE NURSING CRAZE.

Live and Let Live writes: When will it end? Is it just that ladies with large incomes and good homes of their own, who because of the restlessness of the age or some disappointment, should rush into the nursing world and take the bread from those less-favoured gentlewomen who have no resource but to work for their living? When these ladies have, if they must, worked out their probationership, would it not be more graceful to retire from hospital life, and not stop up the better appointments against those who need the home and the money? Would it not be more noble for them to live in their own sphere, give their money to hospitals if they will, but let the more needy gentlewomen live? The nursing world, so far as the better appointments go, is over stocked. Could not rich ladies find some other way of ennobling life than taking bread out of the mouths of others?

A Happy Cottage Nurse writes: I am continually meeting with nurses, in hospital and private, who profess to do the work for the love of it and grumble at the small pay, and at the thoughtlessness of ladies, gardeners, and cooks. Now, sir, I think honour to whom honour is due; i nurses do it purely for love, why not do it gratuitously, taking a small pay to supply themselves with the needful recreations that A. E. S. writes of in this week's Hospital. I am a private nurse and often hold what are called "useful cases" (that is cottage cases). When the patient gets convalescent, the lady that employs me will frequently send me for drives. The carriage I do not expect. In a tax cart I am driven through many country lanes and villages, and at the end of the case the gardener will give me a hamper full of flowers, fruit, and vegetables, with an invitation to spend the day whenever I come.

### Good Temper.

"Cultivate, then, a disposition of kindness and contentment. Let not your own moodiness show out. The patient may have her whims—you may not. Be gentle and soothing in all your acts, and never begrudge your patient the physical charity of a kindly touch, for in many maladies this element of treatment is more important than any combination of drugs."—*Dr. E. L. Keyes.*

### Appointments.

[It is requested that successful candidates will send a copy of their applications and testimonials, with date of election, to The Editor, The Lodge, Porchester-square, W.]

Bradford Infirmary. — Miss Ada Burgin has been appointed charge nurse of the female surgical wards (twenty-five beds) at Bradford Infirmary. Miss Burgin trained for two years at the Grosvenor Hospital for Women, Westminster, and is now just finishing her three years' general training at Guy's. Miss Burgin has been lucky enough to secure an excellent testimonial from Mr. Golding Bird, and other surgeons and physicians have recommended her strongly. We shall be interested to watch Miss Burgin's career, which has commenced under such good auspices.

King's Cross Hospital, Dundee. — Miss Ada Ramsay has been appointed matron of this hospital. Miss Ramsay trained at the Belvidere Fever Hospital, Glasgow, five years ago, and there took a first-class certificate. With a desire to gain knowledge in general nursing, she then went to Perth Infirmary for fifteen months, where she acted in various capacities. Miss Ramsay then took charge of the fever wards of Dundee Infirmary, and lately she has been acting as parish nurse at St. Mary's, Dundee. On May 24, Miss Ramsay was unanimously elected to her present post, which we hope she will find both pleasant and profitable.

### Vacancies.

The following vacancies are announced:

### Matrons.

Atkinson Morley's Convalescent Hospital, Wimbledon, £80.
Fisherton Asylum.
Herts. Convalescent Home, £50.
Harrogate Bath Hospital.
Royal Victoria Hospital, Bournemouth.

### Nurses.

Bedford Nurses' Institute, £26.
Bolton District Nurses' Home, £25.
Bradford Fever Hospital, £23.
Barton-on-Irwell.
Brampton, West Norwood.
City of London Hospital, Victoria Park, £22.
Chelsea Infirmary, £18.
Carmarthen Infirmary, £23.
Epsom New Cottage Hospital, £18.
Hereford Infirmary, £20.
Hull Nursing Institution, £25.
Johnson Hospital, Spalding, £20.
Kingston Home, Hull.
Kingsbridge Union, £25.
Northamptonshire Nursing Institution, two.
Nurses' Institute, Bedford.
Nurses' Home, Norwich.
Providence Home, Nottingham, £25.
Rochdale Infirmary, £22.
St. George's Home, Sheffield.
Stratford-on-Avon Hospital, £20.
St. Cuthbert's Poorhouse, £22.
St. Leonard's-on-Sea Nursing Institution, several.
Woolton Convalescent Institution.
Wirral Children's Hospital, £22.

### District Nurses.

Burton-on-Trent Institution, £25.
Christmas Charity, Gt. Yarmouth, £75.
District Nurses' Home, Bolton.
Nursing Society, Ballymena, £30.

### Probationers.

Burton-on-Trent Institution.

Coldstream Cottage Hospital.

Rivers Street Home, Bath.

### Notes and Queries.

### Queries.

(23) *Egyptian Hospitals.*—Myself and a fellow nurse desire work in a warmer climate, such as Egypt or Italy, to whom should we apply? *Nurse M. B.*

(24) *Catholic Nurses.*—To what institution should a Roman Catholic nurse apply for work? *R. C.*

(25) *Pay Patients.*—What hospitals would take a lady as paying patient? *S. W.*

(26) *Books on Nursing.*—Please advise me what books to read for a nursing examination. *A. G.*

### Answers.

(24) *Catholic Nurses.*—Try the New Bond-street Institution, or Mr. Wilson's Institution, Wimpole-street.

(25) *Pay Patients.*—Apply to Guy's or St. Thomas's, but the charges are high. At the Hampstead Home Hospital they range from 7s. to 105s. weekly. If you like lady doctors, go to the New Hospital for Women.

(26) *Books on Nursing.*—Domville's "Manual," published by Churchill at 2s. 6d., gives a glossary of medical terms, and is therefore very useful. Foster's "Physiology," price 1s., from Messrs. Macmillan and Co., is a good textbook. "Notes on Nursing," by Florence Nightingale; "Medical Nursing," by Dr. Anderson; and "Lectures on Nursing," by Miss Luckes, can also be read with advantage.

